# Effect of Temperature on the Low-Cycle Fatigue Behavior of Polycrystalline TiAl Alloys

**DOI:** 10.3390/ma18133147

**Published:** 2025-07-02

**Authors:** Junyan Zhou, Haochuan Zhao, Pei Li, Henggao Xiang

**Affiliations:** 1 Jiangsu Belight Laboratory, State Key Laboratory of Advanced Casting Technology, Nanjing University of Science and Technology, Nanjing 210094, China; 123116223759@njust.edu.cn; 2 AECC Sichuan Gas Turbine Establishment, Chengdu 610500, China; zhao_haochuan@163.com

**Keywords:** TiAl alloys, polycrystalline, fatigue deformation, temperature effects, molecular dynamics

## Abstract

In this paper, the low-cycle fatigue deformation behavior of polycrystalline γ-TiAl alloys at different temperatures was investigated by molecular dynamics simulations. The results showed that the fatigue process comprises an initial cyclic softening stage followed by saturation, and the stress–strain response of the material shows significant asymmetry. With an increase in temperature, the asymmetry between tensile and compressive stresses gradually decreases, and the amplitude of saturated stress decreases significantly. The decrease in dislocation density leads to the cyclic softening of the alloy, and the evolution of dislocation density is temperature-dependent. The dislocation density first decreases and then tends to be stable, while at 900 °C and 1000 °C, it shows an abnormal trend of decreasing first and then increasing. In addition, microscopic mechanism analysis shows that grain coarsening, dislocation annihilation, and phase instability lead to the cyclic softening of the alloys. The fatigue plastic accumulation at low temperatures is mainly dominated by dislocation slip, while at high temperatures, grain boundary slip gradually replaces dislocation slip and becomes the main deformation mechanism. This work reveals new insights into the mechanical behavior of polycrystalline γ-TiAl alloys under cyclic plasticity and temperature-dependent deformation mechanisms.

## 1. Introduction

γ-TiAl alloys have great application potential in advanced aerospace and automotive fields due to their low density, high specific stiffness, excellent high-temperature creep resistance, and oxidation resistance [1,2,3,4]. Although γ-TiAl alloys have excellent high-temperature properties, these alloys also have problems that are difficult to solve. On the one hand, these alloys have strong room temperature brittleness, poor damage resistance, and a high crack growth rate [5,6]. On the other hand, the oxidation resistance of γ-TiAl alloys at temperatures above 850 °C remains inadequate [7]. Therefore, enhancing the comprehensive mechanical performance of γ-TiAl alloys bears considerable scientific and technical importance.

Fatigue failure is one of the important failure modes of mechanical parts, accounting for more than 90% of mechanical failures [8,9,10]. Previous investigations have extensively focused on elucidating microstructural attributes linked to fatigue-induced mechanical behaviors, with the overarching objective of developing high-precision lifespan prediction models and methodologies for fatigue resistance enhancement. Umakoshi et al. [11,12,13] investigated the plastic anisotropy and fracture behavior of polysynthetically twinned (PST) TiAl single crystals under cyclic loading, focusing on the effects of the lamellar microstructure, strain amplitude, and the angle between the loading axis and lamellar planes. Park et al. [14] studied the low-cycle fatigue behavior of a Ti-46.6Al-1.4Mn-2Mo alloy with a lamellar structure at 800 °C, observing that elevated applied strain ranges induced shifts in the fatigue crack propagation mode. Ding et al. [15] systematically analyzed the microstructure evolution and mechanical behavior of high Nb as-cast γ-TiAl alloys under high temperature and low-cycle fatigue at 850 °C, elucidating the governing mechanisms of phase transformation, lattice strain distribution, and interfacial stresses on fatigue performance. Beran et al. [16] analyzed deformation twin evolution and its effects on mechanical behavior in near-γ-TiAl alloys with 2% Nb during low-cycle fatigue. Their work revealed the competitive mechanisms between twinning and dislocation activity, along with the critical role of twins in cyclic hardening and fatigue life. 

MD simulations have been proven to be an efficient and convenient method for studying the plastic deformation mechanism of nanocrystalline materials [17,18,19,20,21,22]. Nguyen et al. [23] used MD simulations to analyze the dependence of AlCrCuFeNi HEA’s cyclic plasticity behavior on the strain rate and temperature. Zhang et al. [24] explored the fatigue deformation behavior of nickel-based single-crystal superalloys by the molecular dynamics method. Compared with TiAl single crystals, the existence of grain boundaries in polycrystalline TiAl affects mechanical properties, such as plasticity, strength, and fracture of the material, which makes the mechanical properties of polycrystalline TiAl alloys different from those of single crystals [25,26]. For polycrystalline TiAl alloys, the effects of grain size, temperature, and different layered structures on mechanical properties and plastic deformation were discussed. Cao et al. [27] used the MD method to investigate how supersonic fine particle bombardment (SFPB) alters the mechanical properties of γ-TiAl alloys at varying grain scales. Tian et al. [28] analyzed the effects of twin boundary spacing and temperature on nano-polycrystalline TiAl alloys by molecular dynamics simulation. The simulation showed that the difference in dislocation activity under tensile and compressive loads is the cause of material tension–compression asymmetry. Through the combination of experiment and molecular dynamics simulation, Li et al. [29] revealed the strengthening effect and mechanism of gradient TiAl alloys. Li et al. [30] studied the atomic-scale deformation mechanism of polycrystalline TiAl alloys during nanoindentation and found that the layered structure significantly reduces the hardness of the material. 

The evolution of microstructures in polycrystalline TiAl alloys is critically controlled by temperature variations. The existing research focuses on the static mechanical behavior of γ-TiAl alloys, but the temperature dependence of polycrystalline systems under cyclic loading is not clear [31,32,33]. To engineer optimal microstructures and enhance material properties, it is necessary to study the fatigue mechanical response and damage mechanism of polycrystalline TiAl alloys at different temperatures and establish the relationship between fatigue performance and microstructures. It is important to understand the failure behavior of TiAl alloys during service and to explore the anti-fatigue method. In this paper, the fatigue behavior of polycrystalline TiAl alloys at different temperatures was studied by the molecular dynamics method. Through analysis of the cyclic stress response, dislocation density evolution, and shear strain distribution, the deformation mechanism and micro-defect evolution during the low-fatigue process at different temperatures were revealed.

## 2. Modeling and Simulation

**Figure 1:**
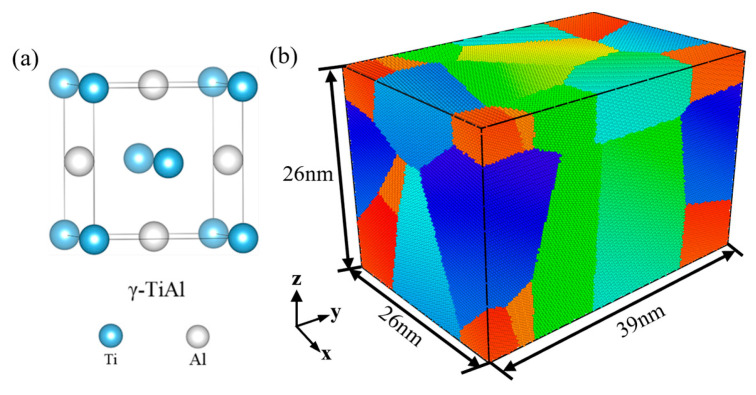
Polycrystalline γ-TiAl initial state (average grain size of 18.5 nm): (**a**) unit cell of γ-TiAl phase and (**b**) grain identity number.

Firstly, the polycrystalline TiAl alloy structure was constructed by the open-source software Atomsk (Version beta-0.13.1) [34]. As shown in Figure 1, the simulation box was 26 × 39 × 26 nm^3^, which contains 1,576,126 atoms. It was composed of 8 grains with random orientation, and the grain size was about 18.5 nm. The interatomic potential of the TiAl embedded atom method (EAM), developed by Zope and Mishin [35], was employed to express the interatomic interactions of the Ti-Al system. MD simulations were performed by the large-scale Atomic/Molecular Massively Parallel Simulator (LAMMPS) (2 August 2023) package. Three-dimensional periodic boundary conditions were applied to eliminate the surface effect. During the equilibrium process, the pressure in all directions was set to 0 by a constant timestep of 1 fs. All simulations were carried out at target temperatures (27, 300, 600, 800, 900, and 1000 °C). Subsequently, the system was subjected to energy minimization by using the conjugate-gradient algorithm [36]. After that, the model was relaxed under the isothermal–isobaric (NPT) ensemble for 50 ps to ensure structural stability at each target temperature. 

**Figure 2:**
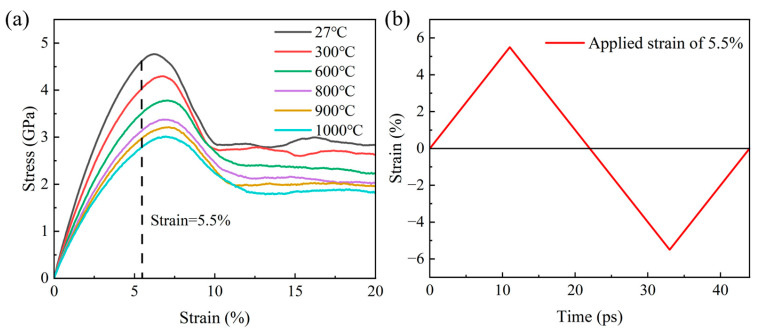
(**a**) Monotonic uniaxial tensile stress–strain curve for polycrystalline TiAl alloys. (**b**) Demonstration of applied symmetric strain cycling.

Before the fatigue test, the equilibrium structure was subjected to uniaxial tensile loading along the *y*-axis at a constant strain rate of 5 × 10^9^ s^−1^ in the NPT ensemble under different temperatures of 27, 300, 600, 800, 900, and 1000 °C to obtain a basic understanding of its mechanical properties. The influence of γ-TiAl on cyclic behavior is largely dependent on temperature and the strain rate. MD simulations have a time scale limitation, and the strain rate is usually much higher than the experimental value. Previous studies have shown that the deformation mechanism of materials is little affected by the strain rate [37,38]. Malti et al. [22] discussed the effects of four loading rates of 5 × 10^8^, 5 × 10^9^, 5 × 10^10^, and 5 × 10^11^ s^−1^ on the mechanical properties of porous Ta/5 wt% Cu alloy. Wu et al. [39] explored the influence mechanism of surface defects on the tensile deformation and fracture of γ-TiAl single crystals at different strain rates (10^7^, 10^8^, 10^9^, and 10^10^ s^−1^). Therefore, the fatigue loading rate of 5 × 10^9^ s^−1^ is reasonable in this paper. Different temperatures have different effects on the fatigue properties of TiAl alloy [40,41]. Thus, the selected temperature range spanned room temperature (27 °C), intermediate temperatures (300–800 °C), and near-melting conditions (900 and 1000 °C). Figure 2a shows the stress–strain curve. According to it, the average flow stress decreases with an increase in temperature. The strain value of ±5.5% was selected for fatigue simulation. Strain-controlled cyclic loading of the polycrystalline TiAl was performed, as shown in Figure 2b. Using the variable loop 100, the program executes 100 loops, and every loop includes two phases of tension and compression. A triangular waveform with a strain ratio of R = −1 was adopted. Finally, the visualization of the simulation results was completed by OVITO [42]. By analyzing the stress–strain hysteresis loop, dislocation evolution (DXA algorithm) [43], and common neighbor analysis (CNA) [44], the deformation mechanism and micro-defect evolution during the fatigue process at different temperatures were revealed.

## 3. Results and Discussion

### 3.1. Cyclic Loading Stress Response

**Figure 3:**
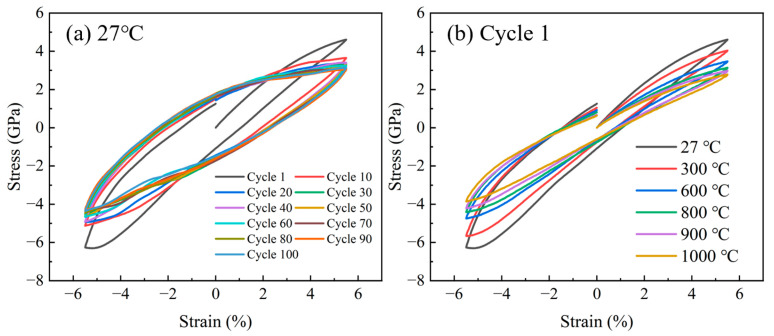
The cyclic stress–strain curves (**a**) at 27 °C. (**b**) The stress–strain curves of the first cycle at different temperatures.

As a vital mechanical metric, cyclic stress–strain behavior deciphers strain-dominated fatigue processes. Figure 3 shows the stress–strain curve of the sample at different temperatures. In Figure 3a, the results indicate the presence of tension–compression asymmetry in the sample during fatigue: the compression stress is much higher than the tension stress. This asymmetry is a typical feature of Cu and Ti alloys [45,46]. The slope of the stress–strain curve decreases with increasing cycles. Both the tensile and compression stages show a significant softening trend. Previous studies have shown that temperature has a significant effect on tension–compression asymmetry [47]. In Figure 3b, comparing the stress–strain curves at different temperatures, it can be found that the difference between the tensile and compressive stresses gradually decreases with rising temperatures. Temperature significantly influences plastic strain energy. The hysteresis loop area peaks at 27 °C. Conversely, elevated temperatures progressively reduce the loop area. This phenomenon demonstrates that the plastic strain energy is larger at room temperature than at high temperatures.

**Figure 4:**
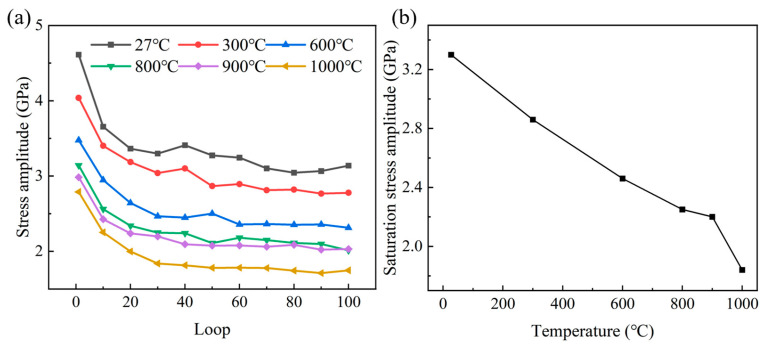
Temperature-dependent stress amplitude: (**a**) cyclic progression at varied temperatures; (**b**) variation in saturation stress amplitude with temperature.

The stress amplitude evolution curves of the polycrystalline TiAl alloys at different temperatures are given in Figure 4a. The results show that the fatigue deformation process of the material exhibits obvious two-stage characteristics at different temperatures: the initial cyclic softening stage and the subsequent fatigue saturation stage. After entering the fatigue saturation stage, the stress amplitude at different temperatures tends to be stable, but it shows temperature dependence. As shown in Figure 4b, the saturation stress amplitude progressively declines with rising temperature, exhibiting maximal reduction at 1000 °C. This temperature effect may be closely related to the thermal activation mechanism of dislocation motion in the material and the dynamic strain aging behavior. Cui et al. [48] carried out low-cycle fatigue tests on TiAl alloys at 750 °C and a strain of ±0.60%; they found that deformation-induced phase transformation is the main mechanism of high-temperature, low-cycle fatigue softening of TiAl alloys. The high-temperature and high-strain-induced α_2_ lamellar degradation and γ grain coarsening are the reasons for the cyclic softening of TiAl alloys.

**Figure 5:**
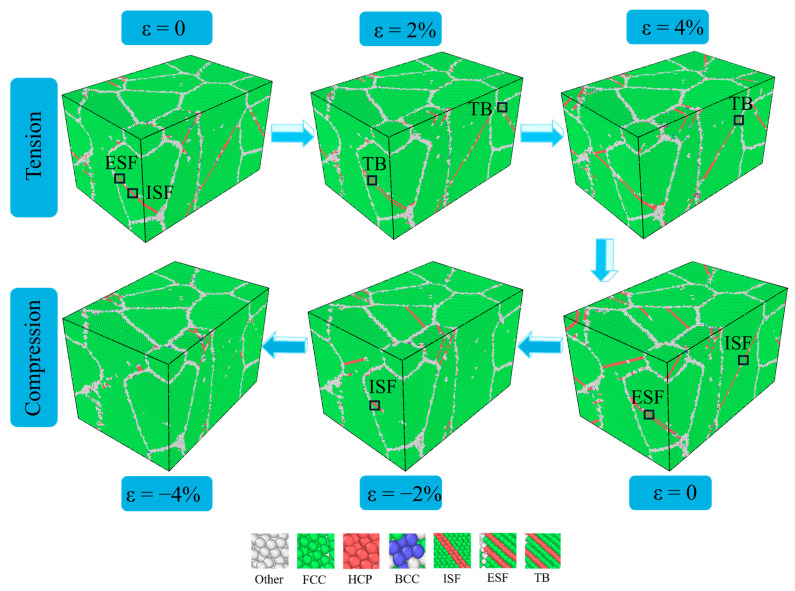
Microstructure evolutions of polycrystalline TiAl alloy under cyclic deformation at 27 °C.

At yielding strain, partial dislocation slip governs incipient plasticity, controlling initial plastic deformation while dynamically generating and removing stacking faults (SFs) and twin boundaries (TBs), which is consistent with the phenomenon observed by Huang and Bowen [49]. During the deformation at 27 °C, as shown in Figure 5, when the strain increases from 0 to 4%, the formed intrinsic stacking faults undergo a complex transformation process, from extrinsic stacking faults (ESFs) or intrinsic stacking faults (ISFs) to TBs inside some grains. When the strain decreases from 0 to −4%, compressive deformation occurs predominantly through dislocation slip. Based on the principle of molecular dynamics, Healy et al. [50] simulated the tension–compression stress asymmetry of nano-iron crystals under cyclic tensile–compression loading. It was found that the deformation mechanisms were different under different conditions: dislocation slip dominated compressive deformation, while twinning prevailed under tensile loading. This difference explained the experimentally observed stress asymmetry of nanocrystals. This is the same as the result we observed. 

**Figure 6:**
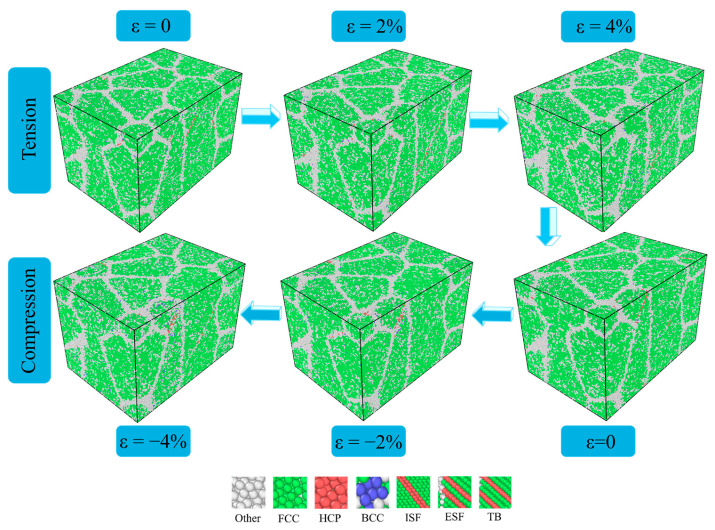
Microstructure evolutions of polycrystalline TiAl alloy under cyclic deformation at 1000 °C.

As shown in Figure 6, at 1000 °C, the number of grain boundary atoms increases, and the grain boundary expands as the grain undergoes plastic deformation. No matter the process of tension or compression, the change in the stacking faults is not obvious. High temperatures accelerate the dynamic recovery process of dislocation climb, cross-slip, and annihilation [51]. These processes help the dislocation structure reach the low-energy state faster and reduce the energy required for dislocation movement in subsequent cycles, especially in overcoming long-range obstacles, thereby reducing the difference in tension–compression asymmetry.

**Figure 7:**
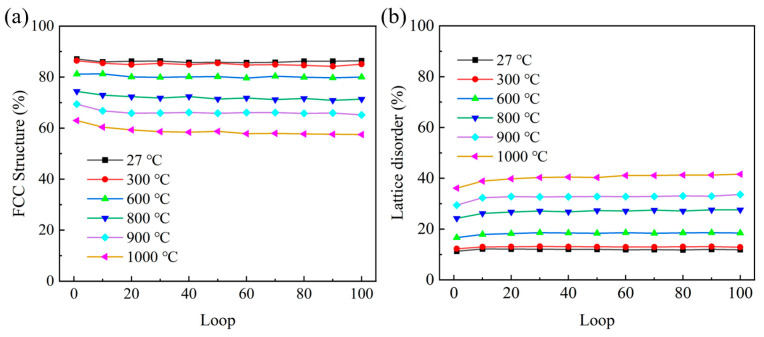
Phase evolution in polycrystalline γ-TiAl alloys during cyclic deformation. (**a**) Fcc structure (**b**) Lattice disorder.

As shown in Figure 6, during the whole cycle at 1000 °C, the stacking faults gradually decrease, and the number of disordered atoms increases. It shows that the high temperature increases the amorphized structure, hinders the movement of dislocations and internal stacking faults, and reduces the asymmetric difference between tension and compression during cyclic loading. This conclusion is proven in Figure 7. It can be seen in Figure 7a that the increase in temperature significantly enhances the decrease in the atomic percentage of the FCC structure. This reduction is attributed to the transformation of the FCC structure into an HCP structure, as well as the development of lattice disorder within the deformation cycle. Meanwhile, as illustrated in Figure 7b, the degree of lattice disorder rises and reaches a stable state with an increasing number of cycles. With cyclic loading, HCP and FCC atoms are transformed into disordered structures. In addition, this phenomenon also indicates that the main plastic deformation mechanism changed at high temperatures.

**Figure 8:**
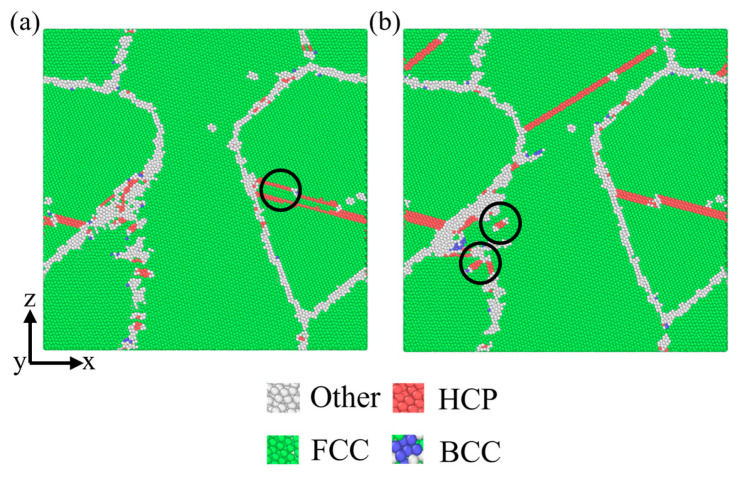
Atomic configurations of polycrystalline TiAl alloy at 27 °C: (**a**) tension loading of ε = 1%; (**b**) compression loading of ε = −1%.

The yield stress of the polycrystalline TiAl alloy under compression is significantly higher than that under tension. This is mainly related to the control mechanism of the initial stage of plastic deformation [52]. Under tensile deformation, twinning is the starting mechanism of plastic deformation. In Figure 8a, it can be seen that there is only one nucleation point of twinning. Under compression deformation, the initial yield is caused by the nucleation and expansion of the partial dislocation, but there are multiple slip systems starting at the same time. As shown in Figure 8b, the start of plastic deformation requires multiple slip systems to reach the critical shear stress at the same time. Moreover, the continuous nucleation of dislocations on the adjacent slip plane forms a complex combination of intrinsic and extrinsic stacking faults, and the stacking fault density is large. Therefore, the compressive yield strength will be higher than the tensile yield strength.

### 3.2. Dislocation Evolution Under Cyclic Loading

**Figure 9:**
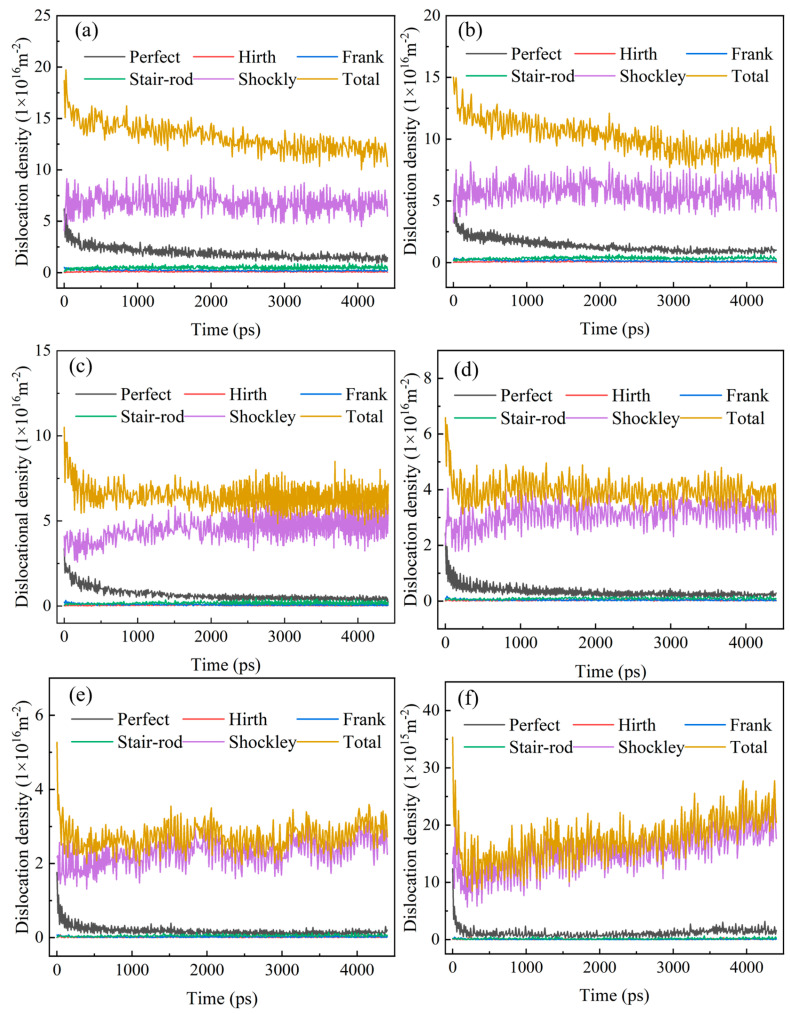
Dislocation density evolution during loading at different temperatures. (**a**) 27 °C, (**b**) 300 °C, (**c**) 600 °C, (**d**) 800 °C, (**e**) 900 °C, (**f**) 1000 °C.

Low-cycle fatigue is a plastic deformation controlled by the formation and movement of dislocations, and dislocation density can reflect the evolution of the microstructure. Therefore, the different types of dislocation density in the model under cyclic loading were quantitatively calculated. The dislocation density evolution of different types at different temperatures is shown in Figure 9. There are mainly two dominant types of 1/6<112> Shockley dislocation and 1/2<110> Perfect dislocation in the model system, and the contribution of other types of dislocation density can be neglected.

During incipient cycling, the dislocation density decreases with the increase in temperature, which is because the increase in temperature significantly enhances the migration ability of dislocations in the grain boundary and its adjacent regions. This enhancement effect facilitates rapid dislocation migration toward grain boundaries and triple junctions, concurrently promoting nucleation of new dislocations at these sites, followed by their absorption, thereby accelerating dislocation annihilation. It is worth noting that the evolution characteristics of dislocation density show significant stage differences before and after reaching the critical saturation value: under all test temperature conditions (except 900 °C and 1000 °C), the dislocation density of each type and the total dislocation density show violent fluctuation characteristics before saturation and tend to be stable after saturation. In particular, it should be pointed out that under the high-temperature conditions of 900 °C and 1000 °C, the evolution of dislocation density presents a unique feature, which is manifested in the evolution trajectory of decreasing first and then increasing. This abnormal phenomenon can be attributed to the effect of dislocation blocking during cyclic loading, which aggravates the formation of dislocation tangles.

### 3.3. Evolution of Cyclic Loading Organization

**Figure 10:**
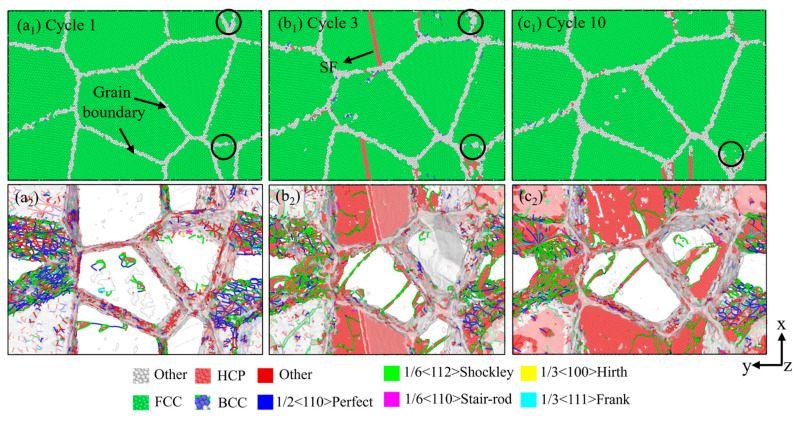
Snapshots at different cycles showing the deformation modes under the tension–compression cyclic loading at 27 °C. (**a_1_**–**c_1_**) the microstructure of model based on CNA analysis (**a_2_**–**c_2_**) the microstructure of model based on DXA analysis.

Figure 10 depicts dislocation and intrinsic stacking fault evolution. It can be observed that cycling at 27 °C induces two visible microstructural evolutions: the decrease in the dislocation density and the annihilation of some grain boundaries. As shown in Figure 10(a_1_–c_1_), with the increase in the cycle period, many of the grain boundaries disappear, leading to an increment in grain sizes. This coarsening reduces the total grain boundary area available to impede dislocation motion, thereby decreasing the alloy’s resistance to deformation, and cyclic softening occurs. In Figure 10(a_2_–c_2_), it can be seen that in the first cycle, there are some dislocation loops in the grains, and the grain boundaries serve as dislocation sources. The stacking fault configuration propagates inward across the grain interior until complete planar development as strain accumulates. The stacking faults are initially initiated from the grain boundary and surrounded by Shockley dislocations. As the number of cycles increases, at the third cycle, the stacking faults gradually expand into the interior of a single grain, and, finally, a completely layered plane is formed inside the grain. At the tenth cycle, the annihilation of dislocations in the grains increases. This density reduction promotes dislocation mobility and makes plastic deformation more likely to occur. Therefore, the yield strength of the material decreases, showing cyclic softening.

**Figure 11:**
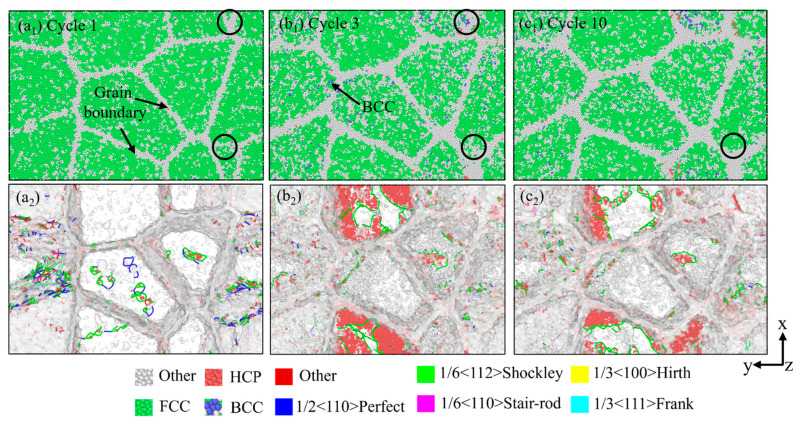
Snapshots at different cycles showing deformation modes under tension–compression cyclic loading at 1000 °C. (**a_1_**–**c_1_**) the microstructure of model based on CNA analysis (**a_2_**–**c_2_**) the microstructure of model based on DXA analysis.

Cyclic loading can induce phase instability; this reduced resistance to dislocation motion results in cyclic softening. In precipitation-hardened alloys, cyclic softening is often caused by the shearing of fine precipitates. Under cyclic loading, dislocations shear these precipitates, reducing their coherence with the matrix and the effectiveness of preventing dislocation movement [53,54]. As shown in Figure 11, in the cyclic loading at 1000 °C, the BBC structure appeared in the third cycle. This phenomenon is due to the fact that severe lattice distortion and local strain synergistically generate critical stress exceeding BCC nucleation thresholds, driving FCC-to-BCC transformation in γ-TiAl. In addition, compared with Figure 10, it can be found that the increase in temperature makes the dislocation density decrease significantly. The decrease in dislocation density reduces the potential barrier for further dislocation motion and makes it easier for plastic deformation to occur [55], thus reducing the resistance of the material to deformation during cyclic loading.

**Figure 12:**
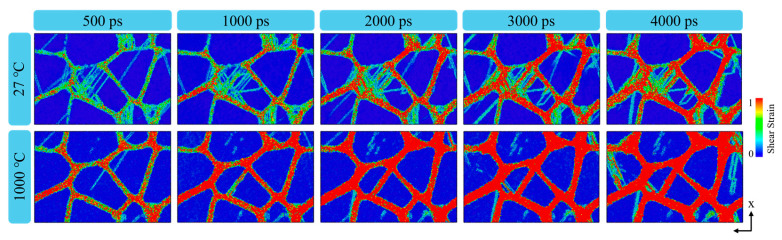
Shear strain distribution of nanocrystalline TiAl at different temperatures of 27 and 1000 °C.

**Figure 13:**
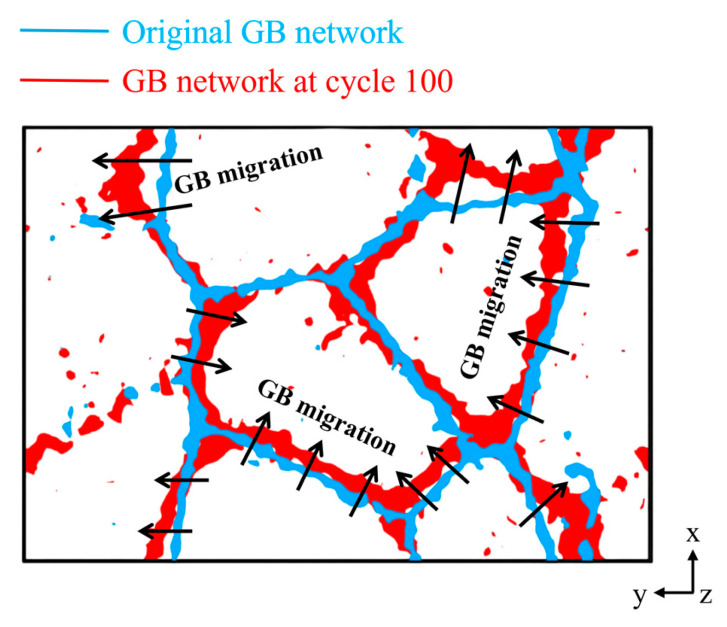
Comparison of the GB network between undeformed and strained states under 1000 °C to demonstrate GB evolution.

In Figure 12, at 27 °C, only intragranular shear bands caused by dislocation interaction are observed in a few grains, while in the remaining grains, local high-strain regions are mainly located near the grain boundaries. Dislocations primarily nucleate at grain boundaries because of the absence of intragranular dislocation sources. The linear displacement trajectory shows that the simple systems in TiAl alloys are activated at low temperature because of the lack of cross-slips hindered by the finite slip system. The limited deformation slip can be activated in a small fraction of grains with a high Schmid factor. Therefore, the plasticity of TiAl alloys decreases with large deformation, and the inhibition of slip by large separation between partial dislocations in low dislocation alloys does not occur in small grains. At this time, plastic deformation is dominated by the dislocation slip mechanism. At 1000 °C, the atomic shear strain near the grain boundary increases significantly. This is due to the weakening of dislocation activity at high temperature, resulting in the transformation of the main deformation mechanism into grain boundary slip [56]. Figure 13 illustrates grain boundary (GB) migration during the initial and final stages of cyclic loading at 1000 °C. A comparison of GB networks between undeformed and strained states demonstrates GB evolution. After 100 loading cycles, the GBs undergo marked migration, as indicated by the arrows.

## 4. Conclusions

In summary, the effect of temperature on the cyclic deformation mechanical behavior of a polycrystalline γ-TiAl alloy was investigated by molecular dynamics simulations. The main conclusions are summarized as follows:

The alloy exhibits significant tensile–compression stress asymmetry under cyclic loading, and the asymmetry gradually decreases with increasing temperature. The fatigue process comprises an initial cyclic softening stage followed by saturation. The amplitude of saturation stress decreases significantly with the increase in temperature, especially at 1000 °C. 

Temperature will affect the characteristics of dislocation density evolution. In the saturation stage, the dislocation density is relatively stable. But under the high-temperature conditions of 900 °C and 1000 °C, the evolution of dislocation density is manifested in the evolution trajectory of decreasing first and then increasing.

Cyclic loading induces grain coarsening and grain boundary annihilation, which reduces the resistance of dislocation motion and leads to cyclic softening. The phase transformation and grain boundary dynamic behavior at high temperatures aggravate the microstructure instability.

Temperature has a significant effect on the deformation mechanism. Dislocation slip dominates plastic deformation at low temperatures, while grain boundary slip gradually becomes the main mechanism at high temperatures, resulting in shear strain concentrated in the grain boundary region.

## Data Availability

The original contributions presented in this study are included in the article. Further inquiries can be directed to the corresponding author.
